# Intestinal Anastomotic Healing: What do We Know About Processes Behind Anastomotic Complications

**DOI:** 10.3389/fsurg.2022.904810

**Published:** 2022-06-07

**Authors:** J. Rosendorf, M. Klicova, I. Herrmann, A. Anthis, L. Cervenkova, R. Palek, V. Treska, V. Liska

**Affiliations:** ^1^ Department of Surgery, University Hospital in Pilsen, Pilsen, Czech Republic; ^2^ Laboratory of Cancer Treatment and Tissue Regeneration, Biomedical Center, Faculty of Medicine in Pilsen, Charles University, Pilsen, Czech Republic; ^3^ Department of Nonwovens and Nanofibrous Materials, Faculty of Textile Engineering, Technical University of Liberec, Liberec, Czech Republic; ^4^ Department of Mechanical and Process Engineering, Nanoparticle Systems Engineering Laboratory, ETH Zurich, Switzerland

**Keywords:** colorectal anastomosis, anastomotic healing, intestinal healing, anastomotic leakage, wound healing

## Abstract

Colorectal surgery has developed rapidly in the recent decades. Nevertheless, colorectal anastomotic leakage continues to appear postoperatively in unpleasant rates and leads to life-threatening conditions. The development of valid complication-preventing methods is inefficient in many aspects as we are still lacking knowledge about the basics of the process of anastomotic wound healing in the gastrointestinal tract. Without the proper understanding of the crucial mechanisms, research for prevention of anastomotic leakage is predestined to be unsuccessful. This review article discusses known pathophysiological mechanisms together with the most lately found processes to be further studied. The aim of the article is to facilitate the orientation in the topic, support the better understanding of known mechanisms and suggest promising possibilities and directions for further research.

## Introduction

Colorectal surgery has developed rapidly in the recent decades. Many new techniques have been introduced lately and the perioperative care keeps changing quite agilely (1–3). Milestones have been taken towards better oncological outcomes, minimally invasive procedures, and improved postoperative quality of life (QoL). Individualized care and the role of the patient’s opinion on their treatment based on their good information and insight into the topic come to the fore. However, this article will discuss the unresolved issues in colorectal surgery, which raise questions not only in this specialization, but across gastrointestinal surgery in its full spectrum.

An essential part of gastrointestinal surgery is a construction of an anastomosis. The concept of resection and reconnection of the hollow parts of the tract is one of the cornerstones of visceral surgery and as such is not expected to be overcome or replaced by other treatment modalities in the foreseeable future.

Just as any other surgical procedure, this one has its specific complications as well. The dreaded anastomotic leak (AL), or dehiscence of the anastomosis, comes to mind first. It is a severe complication that requires a tailored approach depending on its severity. It poses a threat to the patient’s life in the early postoperative period, in many cases requires reoperation, and it is the cause of both longer hospitalizations and reduced postoperative QoL, altogether higher medical care expenses, and according to some studies even worse oncological results (4).

Anastomotic strictures or fistulae are other complications that can occur quite often (5). Strictures, in contrast to leaks, develop over a period of months, so patients are at risk of developing them after they have been placed in home care. In many cases, such stricture may be endoscopically affected, however, a large proportion of patients undergo eventually an additional surgery with resection of the stenotic section of the intestine and are thus again exposed to the risks of major surgical procedure (and risk of stoma for acute procedures) (5, 6). A relatively large number of experimental studies have been performed to find the optimal means to prevent these complications (7, 8). However, few will receive the transfer to clinical medicine.

This article aims to analyze current views on gastrointestinal anastomosis healing and its disorders, to develop the boundaries of these different approaches, the current state of knowledge and to outline areas for further research. The secondary intent of this work is also to draw attention to the fact that, however appealing it may appear to be, hopes for clinical success of current leak prevention research are modest.

## Limitations of Clinical Perspective

The essence of the development of the complications mentioned above are disorders of the healing process. Because the healing process is very complex, the specific cause of the pathology can be located on a wide range of levels (9, 10).

Our current view on the prevention or eventual elimination of these complications relies on the identification of risk factors identified by the correlation of known information about patients with their postoperative course. Based on these data, patients are stratified according to the risk of these complications and appropriate precautions are performed on patients assessed as being at risk (11).

The problem is that we are only able to distinguish AL risk markers for standardly evaluated data. Very broad units such as the presence of immunosuppression, diabetes, old age or male sex thus become markers of high AL risk (Table 1) (11). Such large units in the planning of treatment modalities (Hartman’s resection, protective ileostomies, etc.) are difficult to grasp. Stratification is inaccurate and only some patients benefit from it. We assume AL occurs from a combination of healing abnormalities based on several factors that negatively affect the whole healing process.

**Table 1:** Modifiable and non-modifiable risk factors examples from clinical studies (11–13).

Modifiable	Non-modifiable
Smoking	Male gender
Obesity	Elderly patient
Malnutrition	Emergency procedure
Immunosuppressant treatment	Low anastomosis
Neoadjuvant radiotherapy	Locally advanced tumor
	Diabetes

At present, the process of skin wound healing, including some pathological conditions, is relatively well described (14). However, a similar depth of knowledge is on the digestive tract our utopia. The process is not well documented even in its physiological nature, and certainly the basis of individual pathophysiological deviations is well not researched either (10). Several important points emerge from this statement:

1. Although we know some, we do not know all the risk factors
2. The stratification of patients according to risk is therefore far from being perfect
3. We do not have the opportunity to effectively intervene in the healing process based on its knowledge
4. Applied research in the field of means to prevent anastomotic leakage is in many cases untargeted and ineffective

The degree of unexploredness of such a basic process is an unusual vacuum in scientific knowledge, and the knowledge of the human body.

## Leak Mechanisms Currently in Focus

Before we make the leap into the unexplored, we will introduce the following text with a short discussion about some known pathophysiological mechanisms:

### Anastomotic Leakage and Blood Supply

Blood supply is a key basis not only for the healing process but also for maintaining the vitality of any tissue. Depending on the subtlety of the surgical technique and the condition of the patient’s vascular anatomy and disease, the blood supply to the tissues may be compromised in the terrain of surgery. However, the presence of a sufficient blood supply is a generally valid condition not only to enable any healing process, but also to maintain tissue vitality in any other location. Lacking new information, the frequent research goal is to develop means both for perioperative evaluation of the quality of blood circulation and means to improve the regional blood supply to the anastomosed intestinal tissues. In the first of these, great progress has been made with the introduction of protocols involving the intravenous administration of indocyanine green (ICG) (15). Depending on the quality and speed of ICG distribution to the tissues of the anastomotic intestine, it is then possible to decide on a modification of the resection line. According to published works, individual protocols have the potential to reduce the incidence of anastomotic leakage by tens of percent (16). However, even such a refined technique has not contributed fully to elimination of this complication.

### Anastomotic Leakage and Microbial Infection

Another specific aspect comes to light especially in colorectal surgery. The results of both experimental and some clinical works show the association of the anastomotic leak with infection or colonization of the patient by typical bacterial strains (17). The anastomotic leak caused by the dehiscence of the intestinal anastomosis is certainly an infectious complication, however, the medical society has generally not accepted (at least until recently) the thought that the anastomotic leak is a complication caused by the infection. According to published works, this infection is either a direct cause of leakage development or at least a significant contributor if it develops over an existing healing disorder (17). Pseudomonas Aeruginosa or Streptococcus Faecalis belong among those risk associated pathogens (18, 19). The mechanism of the anastomotic dehiscence lays in the bacterial ability of production of special enzymes consuming newly formed connective tissue of the forming scar – bacterial collagenases (17). These were however identified also in other bacterial strains. If we keep in mind that the basis of collagenase production are bacterial plasmids, which bacteria can share across strains, it may be practically impossible to identify all risky bacterial strains. The second way of negative bacterial influence on the connective tissues of the intestinal anastomosis is mediated by human collagenases, where the bacteria do not produce collagenase itself, but a human collagenase activator, which acts locally by breaking down collagen fibers in a similar way as bacterial collagenases. Bacterial activator of matrix metalloproteinase 9 can play such role (20). During the process of formation the collagen-rich extracellular matrix is degraded by these enzymes. This can lead to mechanical weakness of the anastomosis or even dehiscence (20).

## Emerging Pathophysiological Mechanisms, Processes to be Studied Further

As stated before, today’s knowledge about intestinal anastomotic healing is limited (10). Wound healing is a process probably far more complex than we describe it by today’s view. Many works rely on similarities between the cutaneous wound healing and anastomotic wound healing. This is despite the fact these are completely different organs, located in a completely different environment of the human body. These organs differ in their morphology, representation of individual cell types, blood supply, type of function, etc. While monitoring skin healing is less technically demanding both in the clinical environment as well as in the experiment, direct monitoring of intestinal anastomosis healing inside the abdomen is at least for now, practically impossible. In addition, the intestinal wall consists of several completely different layers, where we can say with certainty that the contribution of each of them to a successful healing process is different, while the essence of the proper function of one of the layers is not to adhere to anything (the mucosa). Pathology in the process of peritoneal healing can form extensive peritoneal adhesions, at the level of the muscular layer, pseudodiverticula may form, and if the process of healing of the intestinal mucosa is altered, fistulas may develop (21). On the other hand, the large intestine is able to heal despite contamination by common feculent flora, while essentially any contamination of a similar type leads to a purulent complication in the skin wound.

The small and the large intestines comprise many different cell types that are also specific for the location on the gastrointestinal tract. The current histological view recognizes in both the small and the large intestine four basic morphological layers: serosa, muscularis, submucosa and mucosa. However, these can be divided into even more units, and even these have their morphological variations depending on the level on the gastrointestinal tract. This situation is the reason why it is so complicated to describe the whole process, including its pathophysiological abnormalities, and why no one has yet been able to describe it in full scale (10).

It is practically impossible to create a comprehensive study monitoring all cell types and their metabolic changes in the healing process at once. Thus, although projects focusing on individual small aspects of the process, such as research into the effects of transient ischemia on peritoneal fibroblast metabolism, have received little attention and often little success in terms of financial support, they are the only means to push forward our current view on the issues of physiology and pathophysiology and the possibility of influencing the healing process in the digestive tract in a targeted matter.

Given the above lack of knowledge, we are not sure which cells, or which intestinal wall layers are the most important for the healing process, or if the interplay of individual layers in the whole process is essential.

The wound healing process is traditionally divided into several overlapping phases for the purpose of simplification: hemostasis, inflammation, proliferation, and remodeling phase (22) by the todays view. The initial three phases form together the acute period which is important for the possible development of anastomotic leakage. However, subtle disbalance can cause problems in the following period resulting in the healing pathologies as anastomotic strictures or fistulae formation.

We propose several issues appearing lately in the literature, that should be studied further for each intestinal layer to resolve some key questions about the healing process. These research topics are just the tip of the iceberg which is the yet to be discovered:

### The Peritoneum

The peritoneum: The healing capacity of peritoneum is enormous (23). Most of the relevant known pathophysiological processes are described in studies focusing on the problematics of postoperative formation of extensive peritoneal adhesions, and not on the problematics of insufficient peritoneal healing. However, both processes start with peritoneal injury followed by inflammation.

A wide range of experimental models were created for the study of peritoneal adhesions, in which not only anti-adhesion agents were systematically verified, but also the very nature of their formation: patient related factors, perioperative factors, influence of surgical techniques on morphology, amount and properties of adhesions (24). The role of molecular factors, cytokines, in the cellular metabolism of peritoneal cells is also being discussed relatively deeply.

Because peritoneal adhesions have been studied extensively, also the peritoneal injury process that precedes the formation of adhesions is well described. The injured surface starts producing a thin fluid which is rich in many proteins and signal molecules as well as inflammatory and other cells (25). This fluid coagulates within 3 h and thus it ensures stable contact of the two peritoneal surfaces. A process of fibrinolysis takes place at the same time and inhibits the formation of adhesion in normal peritoneal healing within the first 72 h after the injury (25). A prolonged persistence (3–5 days) of this coagulated mass is needed for fibroblasts to migrate in it and start producing the extracellular matrix and other substances. This new scaffold is afterwards occupied by mesothelial cells (26, 27). Healthy peritoneum has fibrinolytic activity (prevents obliteration of abdominal cavity in normal circumstances), which can be however decreased in different situations (hypoxia, injury, infection, etc.) leading to adhesion formation (28).

In the formation of peritoneal adhesions, a permanent transformation of peritoneal fibroblasts into so called adhesion fibroblasts was described. It is a change causing increase in proliferation and deposition of collagenous fiber rich extracellular matrix. A variety of signal molecules play their role in regulation of this process but the pathways leading to adhesions formation seem to have common triggers, which are ischemia, hypoxia, and hypercapnia etc. (26, 27, 29). The changes are described as permanent on the cellular level.

The biological role of the peritoneum appears to be relatively clear in the injury: with the highest priority, it is necessary to prevent perforation of the gastrointestinal tract into the free space of the abdominal cavity. Factors such as localized incomplete tissue hypoxia, hypercapnia, or other local markers of cell damage are thus triggers for the proliferation of peritoneum cells and the production of connective tissue to an intense extent, which seems to hastily prevent an acute threat. A long-term disadvantage of the process is that it is a probable cause of over-deposition of collagen-rich connective tissue, for example in the construction of gastrointestinal anastomosis, and thus contributes to stricture formation.

Dysregulation of these molecular factors has been described in the literature to be triggered by local ischemia: Tissue plasminogen activator (tPA), Transforming growth factor-β1 (TGF-β1), Tumor necrosis factor α (TNF-α), Interleukin 6 (IL-6), Matrix metalloproteinases (MMPs), Cyclo-oxygenases (COX) (30–38). However, the aim of this article is not to describe individual events that are relatively complex, so we recommend the cited literature for a deeper study.

### The Muscular Layer

Isolated defects of muscular layer can be seen in imperfectly healed anastomoses as fistulae or can form a pseudodiverticula when the peritoneal surface maintains integrity. However, fistulas are more suspicious of being a mucosal healing imperfection. The hypertrophy of the muscular layer is not usually recognized in intestinal anastomoses but often in patients with chronic inflammatory bowel disease, where it is responsible for intestinal wall thickening and formation of strictures. A state of chronic inflammation with constant production of inflammatory signal molecules and infiltration by inflammatory cells causes a change of metabolism of smooth muscle cells (SMCs). The SMCs gain proliferative ability and start producing extracellular matrix (ECM) (39, 40). The role of ischemia on the SMCs was not described though, and the effect of inflammatory cytokines on SMCs in the process of anastomotic healing is unknown as well. This is certainly material for further basic research.

### The Submucosa

The submucosa is the layer that is known to be the mechanically strongest. The fact that it contains a lot of collagen rich ECM makes most of clinicians suppose it is the most important layer for optimal anastomotic healing (41). Yet it is not known whether it really is activated in the process of anastomotic healing in sufficient amount to regain mechanical strength in time. And moreover, there is yet no proof suggesting that mechanical strength can be relied on in intestinal anastomosis and there is a probability that there is no link between anastomotic leakage risk and the mechanical strength. The metabolism of the submucosal tissue has not been studied thoroughly neither in normal circumstances nor after injury. Further basic research needs to be conducted urgently.

### The Mucosa

Intestinal epithelial cells belong among the most rapidly proliferating cells in human body. In normal situation thousands of cells are scrubbed from the mucosal surface by food passage every day. Mature enterocytes however do not have any proliferative capacity and so the mucosal renewal depends on proliferation and differentiation of stem cells located in intestinal crypts. They are responsible for re-epithelization when it comes to anastomotic healing as the mature enterocytes cannot regain this ability. Epithelial mesenchymal transition is a healing associated cellular transformation responsible for de-differentiation of mature epithelial cells and for their regain of the proliferative activity. Newly formed epithelial cells keep covering denuded luminal surface, but do not adhere to epithelized surfaces (One of the basic biological assumptions is that the mucosa must not grow another mucosa surface to surface in order to avoid loss of intestinal lumina). It has been described that the process of superficial proliferation is probably responsible for fistulae formation in patients with anorectal inflammatory bowel disease (42–44), whether the process is behind fistulae formation in case of intestinal and bowel anastomosis is not confirmed, but the mechanisms could be similar. The epithelium is not considered to participate in formation of anastomotic strictures, but not enough research has been conducted to rule out even this assumption.

An interesting view that has not been sufficiently explored is also the importance of barrier function of the mucosa and its loss from intestinal injury, the process of its regeneration, the factors that affect it, and last but not least, how this loss affects the metabolism of the remaining gastrointestinal wall. The basis for the loss of this function is, among other mechanisms, a disorder of tight junctions between enterocytes. It occurs, for example, in septic conditions, where it is another probable contributor to the healing disorder (45). The barrier function suffers also during diarrheic diseases and can be altered also by aggressive laxatives that are used for mechanical bowel preoperative preparation (46).

## Discussion

At present, we have the advantage of the existence of advanced laboratory methods that allow us to observe both metabolic and proliferative changes of individual cells of the gastrointestinal wall, as well as their dynamics and mutual interaction. It is necessary to maximize the use of these auxiliary methods in combination with a clinically relevant experimental model of gastrointestinal healing, both in physiological conditions and to compare these processes with processes taking place in the presence of pathological changes, factors negatively affecting the healing process.

There are many unanswered questions in the process of gastrointestinal healing. Only a thorough research of partial processes, changes at the level of cellular metabolism, at the level of individual layers of the gastrointestinal tract wall, the dynamics of these processes and their interactions under both physiological and pathophysiological conditions can contribute to advances in clinical visceral surgery and other targeted prevention of anastomotic complications including leaks and stenoses. Even though new methods and techniques are emerging in colorectal surgery, the anastomotic leakage continues to haunt us. New technologies allow us to create new kinds of materials for both local or systemic treatment, but not knowing the physiological process and its pathological changes means not knowing what we treat.

## Author Contributions

Conceptualization: J.R., V.L., V.T.; methodology: I.H., L.C., writing original draft: J.R., M.K., formal analysis: V.L., A.A., R.P., V.T. All authors contributed to the article and approved the submitted version.
